# A pilot study to determine the feasibility of a cluster randomised controlled trial of an intervention to change peer attitudes towards children who stutter

**DOI:** 10.4102/sajcd.v65i1.583

**Published:** 2018-07-18

**Authors:** Rizwana B. Mallick, Lehana Thabane, A.S.M. Borhan, Harsha Kathard

**Affiliations:** 1 Department of Communication Sciences and Disorders, University of Cape Town, South Africa; 2 Department of Health Research Methods, Evidence and Impact, McMaster University, Canada; 3 Department of Health and Rehabilitation Sciences, University of Cape Town, South Africa

## Abstract

**Background:**

While randomised controlled trials (RCTs) are considered the gold standard of research, prior study is needed to determine the feasibility of a future large-scale RCT study.

**Objectives:**

This pilot study, therefore, aimed to determine feasibility of an RCT by exploring: (1) procedural issues and (2) treatment effect of the Classroom Communication Resource (CCR), an intervention for changing peer attitudes towards children who stutter.

**Method:**

A pilot cluster stratified RCT design was employed whereby the recruitment took place first at school-level and then at individual level. The dropout rate was reported at baseline, 1 and 6 months post-intervention. For treatment effect, schools were the unit of randomisation and were randomised to receive either the CCR intervention administered by teachers or usual practice, using a 1:1 allocation ratio. The stuttering resource outcomes measure (SROM) measured treatment effect at baseline, 1 and 6 months post-intervention overall and within the constructs (positive social distance, social pressure and verbal interaction).

**Results:**

For school recruitment, 11 schools were invited to participate and 82% (*n* = 9) were recruited. Based on the school recruitment, *N* = 610 participants were eligible for this study while only *n* = 449 were recruited, where there was *n* = 183 in the intervention group and *n* = 266 in the control group. The dropout rate from recruitment to baseline was as follows: intervention, 23% (*n* = 34), and control, 6% (*n* = 15). At 1 month a dropout rate of 7% (*n* = 10) was noted in the intervention and 6% (*n* = 15) in the control group, whereas at 6 months, dropout rates of 7% (*n* = 10) and 17% (*n* = 44) were found in the intervention and control groups, respectively. For treatment effect on the SROM, the estimated mean differences between intervention and control groups were (95% Confidence Interval (CI): -1.07, 5.11) at 1 month and 3.01 (95% CI: -0.69, 6.69) at 6 months. A statistically significant difference was observed at 6 months on the VI subscale of the SROM, with 1.35 (95% CI: 0.58, 2.13).

**Conclusion:**

A high recruitment rate of schools and participants was observed with a high dropout rate of participants. Significant differences were only noted at 6 months post-intervention within one of the constructs of the SROM. These findings suggest that a future RCT study is warranted and feasible.

## Introduction

### Feasibility of randomised controlled trials

Among the various levels of evidence that are valuable in clinical practice, the randomised controlled trial (RCT) is regarded as the gold standard because of its design strength (Evans, 2003) and its power to draw conclusions (Oakley, Strange, Bonell, Allen, & Stephenson, 2006; Sibbald & Roland, 1998). Because of the large-scale nature of the RCT and its stringent design, there are financial, resource and time implications that require careful consideration before one can be conducted. Consequently, the literature recommends that it is vital to first conduct a comprehensive pilot study to determine feasibility and improve the validity and statistical power of a future RCT (Evans, 2003; Leon, Davis, & Kraemer, 2011; Oakley et al., 2006; Shanyinde, Pickering, & Weatherall, 2011).

It is further reported that the procedural rigour of the study is as important as the treatment effect benefit when evaluating the feasibility of an RCT (Oakley et al., 2006). Procedural aspects and treatment effect are weighted equally in importance to determine feasibility of an RCT, as this approach focuses both on the process of implementing and intervention as well as the outcome (determining the treatment effect) (Evans, 2003; Leon et al., 2011; Oakley et al., 2006; Shanyinde et al., 2011). Therefore, this study aimed to determine the feasibility of a future RCT by assessing two key components, the recruitment of schools and participants and dropout rate of participants, as well as the potential treatment effect of a classroom-based stuttering intervention.

The process evaluations of a study are specifically recommended in longitudinal studies, such as this one, where repeated measures occur (Oakley et al., 2006) at baseline, 1 and 6 months post-intervention. These process evaluations identify any organisational challenges and changes that are required (Akobeng, 2005; Bowen et al., 2009; Kingston, 2004; Oakley et al., 2006; Thabane et al., 2010) to minimise potential flaws or bias (Currie, Seaton, & Wesley, 2009; Downs & Black, 1998; Lancaster, Dodd, & Williamson, 2002; Oakley et al., 2006). The loss of participants is also reported as probable, particularly in longitudinal studies (Keyzer et al., 2005; Morton, Cahill, & Hartge, 2005). Several aspects of process evaluations exist; this study focused on recruitment (school and participant) and dropout rate of participants because of the longitudinal nature of this study. The dropout rate was also selected, as the dropout rate of participants can result in incomplete or missing data (Fitzmaurice, 2003). It may also result in a population no longer being representative, thus reducing the statistical power and validity of a study (Toerien et al., 2009). This was clearly observed in a previous preliminary classroom-based stuttering intervention study (Badroodien et al., 2011).

At present, there are no documented feasibility studies in South Africa within the domain of classroom-based intervention, which is essential for planning of future large-scale studies. Comment on feasibility is also important, as research within the school context is challenging. Common challenges relate to procedural aspects and treatment effect such as consent, participation and ethical concerns resulting from the vulnerable nature of conducting research with children. While it could be argued that this is the case for all studies, the complexity of school research adds to the level of difficulty that is often experienced when conducting school-based research.

In terms of treatment effect, it is essential to also determine the potential treatment effect in a pilot study design prior to conducting an RCT. Lancaster et al. (2002) reported that an intervention may not appeal to all and thus acceptability of the intervention should be studied as part of determining treatment effect. Questions around whether the intervention works and to what extent, whether the intended outcomes are achieved, its benefits and harms (including for whom) may also be answered through the study of potential treatment effect of an intervention (Evans, 2003). In addition to knowing whether there is any potential shift in treatment effect, the inclusion of treatment effect measures may also determine when treatment effect should be measured, specifically, which time interval shows a greater shift, if any. A pilot study is therefore required so that when an RCT is conducted, there are findings of a pilot study to show that time, resources and money can be justifiably invested into doing a RCT study. For this reason, it is critical to focus on both aspects, procedural and treatment effect, to accurately inform the feasibility of an RCT.

### Classroom-based intervention

Stuttering, a communication disorder, presents with personal and social implications (negative self-perceptions, teasing and bullying), often occurring at primary school (Dijkstra, Lindenberg, & Veenstra, 2008; Murphy, Yaruss, & Quesal, 2007; Swearer, Espelage, Vaillancourt, & Hymel, 2010). Despite the lack of training and resources to address communication difficulty and disabilities reported by teachers (Penn, Watermeyer, & Schie, 2009), teacher involvement and training has been found to prevent teasing and bullying (Blank et al., 2009). Classroom-based intervention has therefore been advocated as a strategy to improve peer attitudes (Langevin, 2009; Merrell, Gueldner, Ross, & Isava, 2008; Murphy et al., 2007) internationally (Langevin, Bortnick, Hamer, & Wiebe, 1998) and in South Africa (Branfield et al., 2015; Farelo et al., 2015; Hobbs et al., 2016).

Internationally, persistent reports of teasing and bullying of children who stutter (CWS) led to the development and study of the Teasing and Bullying (TAB) resource, a classroom-based intervention in Canada (Langevin, 1998). The TAB was created on the basis that attitude is learnt and can be changed (Foster, 2006). The TAB was found useful and feasible in targeting negative peer attitudes in a pre- and post-intervention study (over three to four temporal periods), without a control group, among Grades 3–6 learners (Langevin, 1998, 2009; Langevin & Prasad, 2012). However, it was not suitable for the South African population, linguistically or culturally. Furthermore, the lack of a control group as a methodology does not align with the planning of a future RCT.

The TAB resulted in the development of the Classroom Communication Resource (CCR) intervention for South Africa, the intervention being subjected to testing in this study. It was required specifically in South Africa because of the prevalence of teasing and bullying and requests from teachers for support (Abrahams, Harty, St Louis, Thabane & Kathard, 2016; Hobbs et al., 2016). The focus of the CCR intervention is to target peer attitudes. The example of stuttering and communication is used in the CCR intervention but it can be extended to target difference, acceptance and teasing and bullying. The CCR intervention is a classroom-based resource that is administered by teachers, as the communication partner.

The CCR intervention was studied and developed through small-scale studies by the University of Cape Town between 2009 and 2014 (Badroodien et al., 2011; De Grass et al., 2010; De Freitas, Geben, Parusnath, Relleen, & Van den Berg, 2012; Filies, Hartley, Kaplan, & Pettit, 2009; Kathard et al., 2014; Walters, 2014). In 2014, Kathard et al. studied the attitudes of Grade 7 peers of CWS at pre-intervention and 1 month post-intervention, where the CCR was administered by teachers to intervention groups only. The stuttering resource outcomes measure (SROM) was used to measure attitudes at pre-intervention and 1 month post-intervention in control and intervention groups in the areas of pro-social behaviours – positive social distance (PSD), verbal interaction (VI) and social pressure (SP). The results of the study yielded minimal positive effects at 1 month post-intervention (Kathard et al., 2014). Kathard et al. (2014) subsequently recommended a large-scale study to explore peer attitudes over time, as they reported uncertainty around time intervals to determine treatment effect. This study therefore aims to build on the findings and recommendations of Kathard et al. (2014) by exploring the treatment effect at both 1 and 6 months post-intervention as well as procedural aspects to assist with future planning of an RCT.

## Aim

The aim of this study was to determine the feasibility of an RCT through conducting a pilot study.

## Objectives

The study had two objectives:

- Primary objective: to determine the recruitment rates of schools and participants and the dropout rate of participants
- Secondary objective: to determine the treatment effect of attitudes towards stuttering among Grade 7 students based on the SROM and its subscales – the PSD, VI and SP.

## Methods

### Study design

A pilot, cluster, stratified RCT design was used, where schools were the unit of randomisation. The cluster stratified RCT design was emulated using a pilot study design to accurately comment on the feasibility of a future RCT. The schools were stratified into two quintile groups (lower vs. higher) and randomised to receiving the CCR intervention or usual practice, using a 1:1 allocation ratio.

### Participants

The eligibility criteria included Grade 7 participants, aged 11 years and older, in mixed-gender schools where the language of learning and teaching was English. The participants attended public primary schools in the lower (two and three) and higher quintiles (four and five). Quintiles were included to ensure a representative sample was included. The schools were situated in the Western Cape metropolitan urban area in South Africa. Participants were not financially compensated in any way. All participating schools were provided with their own copy of the CCR intervention. Schools could have CWS in the classroom; however, once CWS were identified they were approached to obtain consent to determine if the study could be conducted in their school. Exclusion criteria included participants aged younger than 11 years from same-sex schools and schools within Quintile 1.

### Sample size

A total sample size of *n* = 401 was included where *n* = 149 children were included in the CCR intervention and *n* = 252 children in usual practice (control group). A minimum sample size of *n* = 192 was recommended based on power analysis calculations from previous studies in the project stream using observations of treatment effects and mean differences (Badroodien et al., 2011; Kathard et al., 2014; Walters et al., 2014). This study aimed to include a minimum of *n* = 384 (Walters, 2014).

### Intervention

#### Classroom Communication Resource intervention

The CCR intervention included three key components, namely a social story, role play and teacher-led discussion. The teacher read the social story to his or her class. Once the story was complete, the teacher selected participants to act out the role play. The purpose of the role play was to emphasise the story but also to provide participants with a first-hand account of how the characters of the story may have felt. Finally, the teacher facilitated the discussion by using the guidelines in the CCR intervention. The discussion aimed to promote acceptance of diversity and difference related to stuttering, communication and generally, as well as discussions around teasing and bullying and how this related to what was happening at each school.

The CCR intervention is considered self-sufficient for the most part. However, the teacher was provided with basic training on how to administer the CCR intervention. The focus on training was placed on the discussion aspect of the intervention, as many teachers had queries and concerns about how to best administer this section. In doing so, the CCR could be considered a supported classroom-based intervention that was used as a single-dose intervention. The researchers observed, without interference, the administration of the CCR intervention. The CCR intervention was only administered in the intervention groups, while control group teachers continued with their teaching without drawing attention to stuttering in any way. Any questions that were asked after the intervention were to be answered and recorded by the teachers.

### Outcomes measure

#### Procedural aspect

The recruitment rate described the number of schools that were invited compared to those who agreed to participate during the recruitment phase of this study. This was described at a school-level, as this is how participants were initially recruited. Thereafter individual recruitment was described in terms of those recruited from the eligible sample (based on school recruitment). The dropout rate described the loss of participants at baseline, 1 and 6 months post-intervention because of the longitudinal nature of this study.

#### Treatment effect: Stuttering resource outcomes measure

This study is concerned with the observation of a positive shift in the treatment effect (magnitude and direction) at 1 and 6 months post-intervention from baseline. The treatment effect was commented on using the global and sub-scale scores on the SROM. The SROM consisted of 20 questions making use of a Likert scale. The SROM sub-scales, including PSD, VI and SP, are psychometrically approved constructs (Walters, 2014).

The SROM was developed on the Peer Attitudes towards Children Who Stutter (PATCS). The PATCS was developed by Langevin (1998) in Canada while the SROM was developed for the South African population. The criterion reliability of the PATCS (Langevin, 2009; Langevin, Kleitman, Packman, & Onslow, 2009) and SROM was met (Walters, 2014).

#### Sampling and enrolment

Once-off randomised sampling took place to track participants from baseline, to 1 and 6 months post-intervention. Continuous sampling was therefore not practical.

#### Data collection procedure

Upon obtaining the relevant consent and assent, all participants viewed a video of a CWS. The participants were all asked to complete the SROM at baseline. Thereafter the teachers in the intervention groups received training, over a 60–90 min session, to administer the CCR intervention. The CCR intervention was then administered by the teacher to the participants in the intervention groups. No intervention took place in the control group. At 1 and 6 months post-intervention, all participants completed the SROM. Thereafter the control group teachers were provided with a copy of the CCR as well as teacher training.

## Statistical analysis

The procedural aspects are calculated as follows:

- The school recruitment rate was determined by examining how many schools were invited and agreed to participate. Individual recruitment was similarly reported.
- The dropout rate was calculated into a percentage value at each time interval (from baseline, 1 month and 6 months post-intervention). It was reported as it is a common occurrence within the school setting and accounts for the participant numbers noted in this study. The inclusion of this information is essential for future planning of an RCT.

The treatment effect is calculated as follows.

Each participant’s SROM scores were captured in Microsoft Excel, and R Studio version 1.0.143 (http://www.studio.com) software was used to analyse the data. Information such as knowing someone who stutters and scores according to gender was not reported on, as no significant differences were noted in previous studies (Badroodien et al., 2011; Kathard et al., 2014; Walters, 2014). The random effect model was used to assess the intervention at 1 and 6 months post-intervention and was also used to account for potential correlation among the outcomes from schools. Additionally, the intra-school correlation coefficient (ICC) was reported on. An estimate of the ICC difference between groups, 95% confidence interval (CI) and associated *p*-values (to three decimal places, with those less than 0.001 reported as *p* < 0.001) was reported. The analyses were adjusted for the stratification covariate quintile. Sensitivity analysis was also performed to examine the treatment effect using linear regression, which did not account for the potential correlation among the outcomes from schools.

## Ethical consideration

Ethical approval was obtained from the University of Cape Town Health Sciences Human Research Ethics committee (510/2013). Thereafter, permission was provided by the Western Cape Education Department. Consent and assent were obtained from schools, principals, parents and participants. The ethical principles of autonomy, confidentiality, beneficence, non-maleficence and distributive justice were upheld at all times, as stipulated by the Declaration of Helsinki (Williams, 2008).

## Results

### Feasibility aspects

#### Recruitment rate

A total of 11 schools were invited, 10 schools responded to the invitation to participate, nine schools accepted the invitation and only eight participated in this study, as one school withdrew from the study. The recruitment rate was therefore 82%, as 9 out of the 11 schools invited agreed to participate in this study. Based on the school recruitment, *n* = 610 participants were eligible for this study while only *n* = 449 were recruited, where there were *n* = 183 in the intervention group and *n* = 266 in the control group as a result of absenteeism and not providing consent.

#### Dropout rate

The dropout rate in the intervention group at baseline was 23% (*n* = 34) and 6% (*n* = 15) in the control group because of consent not being provided and absenteeism. At 1 month post-intervention, a dropout rate of 7% (*n* = 10) was noted in the intervention and 6% (*n* = 15) in the control. At 6 months post-intervention, dropout rates of 7% (*n* = 10) and 17% (*n* = 44) were noted in the intervention and control groups, respectively (Table 1).

**TABLE 1:** Dropout rate at baseline, 1 and 6 months.

Time point	Dropout			

	Intervention (*n* = 149)		Control (*n* = 252)	

	*n*	%	*n*	%
Baseline	34	23	15	6
1 month	10	7	15	6
6 months	10	7	44	17

### Preliminary estimates of treatment effect

A total of *n* = 401 were analysed, with *n* = 149 in the intervention group and *n* = 252 in the control group, with 42% male in each group. A total of eight clusters (schools) were analysed, equally divided in terms of quintile to ensure a more representative sample. The baseline SROM score mean was 73.17 (SD 12.05) in the intervention and 71.48 (SD 12.80) in the control group. The baseline characteristics are shown in Table 2.

**TABLE 2:** Baseline characteristics of study participants by study group.

Variable	Intervention (*n* = 149)						Control (*n* = 252)					

	*n*	%	Mean	SD	Min	Max	*n*	%	Mean	SD	Min	Max
Number of clusters	4	-	-	-	-	-	4	-	-	-	-	-
Cluster size	-	-	37	-	25	57	-	-	63	-	25	141
Gender: male	63	42	-	-	-	-	105	42	-	-	-	-
**Baseline score**													
SROM	-	-	73.17	12.05	-	-	-	-	71.48	12.80	-	-
PSD	-	-	38.65	7.63	-	-	-	-	38.21	7.55	-	-
SP	-	-	19.89	3.68	-	-	-	-	19.15	4.14	-	-
VI	-	-	14.62	3.28	-	-	-	-	14.12	3.37	-	-

SROM, Stuttering resource outcomes measure; PSD, positive social distance; SP, social pressure; VI, verbal interaction.

**FIGURE 1:**
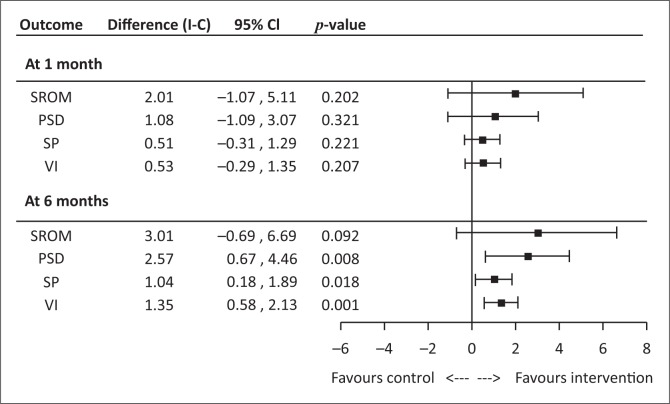
Forest plot of treatment effect at 1 and 6 months on the stuttering resource outcomes measure and its subscales positive social distance, social pressure and verbal interaction (*p* = 0.001).

As shown in Figure 1, the key findings indicate no significant differences (95% CI) in the SROM with 2.01 (–1.07, 5.11) at 1 month post-intervention and at 6 months post-intervention with 3.01 (–0.69, 6.69). Findings showed no significant differences at 6 months in the constructs of PSD, 2.57 (0.67, 4.46), and SP, 1.04 (0.18, 1.89). The only significant difference noted was at 6 months within the construct of VI, with 1.35 (0.58, 2.13).

A sensitivity analysis was also conducted ignoring clustering, which showed similar results as shown in Table 3. Table 3 includes the estimated differences between the intervention and control groups, along with 95% CIs and *p*-values, adjusted for quintile, for the outcomes SROM, PSD, SP and VI. Sensitivity analysis, ignoring clustering, showed similar results at 1 month post-intervention with 2.01 (–1.09, 5.12) and 6 months post-intervention with 2.46 (–1.05, 5.98).

**TABLE 3:** Sensitivity analysis of treatment effect at 1 and 6 months on the stuttering resource outcomes measure sub-scales (*p* = 0.001).

Variable	Outcome	Method	Difference (I-C)	95% CI	*p*-value	ICC
At 1 month	SROM	Random effects	2.0100	−1.07, 5.11	0.2020	< 0.001
		Linear regression	2.0140	−1.09, 5.12	0.2030	-
	PSD					
		Random effects	1.0800	−1.09, 3.07	0.3210	0
		Linear regression	0.9900	−1.10, 3.08	0.3530	-
	SP					
		Random effects	0.5100	−0.31, 1.29	0.2210	0
		Linear regression	0.5000	−0.30, 1.29	0.2210	-
	VI					
		Random effects	0.5300	−0.29, 1.35	0.2070	< 0.001
		Linear regression	0.5300	−0.30, 1.36	0.2110	-
At 6 months	SROM	Random effects	3.0100	−0.69, 6.69	0.0920	0.015
		Linear regression	2.4600	−1.05, 5.98	0.1690	-
	PSD
		Random effects	2.5700	0.67, 4.46	0.0076	0
		Linear regression	2.5700	0.66, 4.47	0.0085	-
	SP					
		Random effects	1.0400	0.18, 1.89	0.0180	0
		Linear regression	1.0362	0.17, 1.90	0.0186	-
	VI					
		Random effects	1.3500	0.58, 2.13	0.0010	0.043
		Linear regression	1.2100	0.46, 1.96	0.0017	-

ICC, Intra-school correlation coefficient; SROM, stuttering resource outcomes measure; PSD, positive social distance; SP, social pressure; VI, verbal interaction.

## Discussion

### Generalisability of findings

It should be noted that the findings of this study reflect schools in the Western Cape, South Africa, from Quintiles 2, 3, 4 and 5. Findings should, therefore, be interpreted with caution when considering other provinces within South Africa.

### Feasibility aspects

Several challenges were encountered during this study, despite the anticipation of some general challenges that often arise during school-based research. It could be argued that all researchers experience varying degrees of difficulty with conducting research, while the complexity of school research added to the level of difficulty that was experienced in this study. The common challenges were experienced, such as consent and participation, which affected the recruitment of participants.

The results indicate that the recruitment rate was high because schools were approached early in the year and could thus foresee making time available for the researchers, showing that school recruitment may be a feasible method of initial recruitment. While there is disparity in the numbers of control versus intervention groups, it should be noted that this was as a result of consent not being provided and absenteeism, all factors out of control of the researcher. Furthermore, this was taken into account when interpreting the findings of this study. Once the challenge of school recruitment was overcome, the researcher faced difficulty with recruiting individual participants. Based on the eligible participants from school recruitment, far fewer participants were recruited, as a result of poor consent. It was challenging because the researcher relied on schools, principals, teachers, parents and participants to provide permission, consent and assent required for recruitment, while the researcher only had access to principals and some of the teachers. Because of the strict design of RCTs, the study will only be successful should schools agree to participate and facilitate return of consent forms from parents. Once participants were recruited, the next challenge was to retain participants and prevent a large dropout of participants to ensure power analysis of this study. It was reported by schools that clearer communication is required. It is, therefore, vital that, in future, schools and teachers are made explicitly aware of the time commitments required of them so that they may make an informed decision as to whether they are able to participate in a future study and not experience the burden of participating in a study.

Upon discovering that data were to be collected at three separate intervals (baseline, 1 month and 6 months) in addition to another visit to the school where the teacher administered the CCR, schools expressed anecdotally great concern around the time commitments required of them. As a result, a dropout of participants was noted over time as well as difficulty arranging for data collection dates. This is commonly reported in longitudinal studies (Galea & Tracy, 2007). Schools became increasingly hesitant to commit to an additional suitable time for data collection at 6 months post-intervention when compared to 1 month post-intervention. Schools felt that they had already provided this study with a substantial amount of time and no longer perceived their participation to be beneficial, which is a common determinant of a dropout rate (Galea & Tracy, 2007; Lundberg, Thakker, Hällström, & Forsell, 2005). Schools requested that, in future, data be collected over fewer time periods (i.e. perhaps only at 6 months) with fewer visits to the school. The researcher should also be understanding of the schools, their processes and preferred methods for participating and that all schools are different. Researchers should also display an awareness of the sacrifices that schools make to be able to participate in research studies. Because of the time constraints, schools asked that the research take place at the end of the school term. However, it meant that many participants were absent from school at the end of the term after completing their academic testing. This is reported by teachers in this study to be a common occurrence, as no new work was being taught at school.

In terms of organisation, early planning and scheduling, logistically it was challenging to find suitable times for data collection, given the pre-existing busy academic calendar. Schools found the research time-consuming and reported that they would not have committed to it had they realised the extent of the time needed to dedicate to this study. Consequently, there were serious implications in terms of motivation to participate and the relationship between the researcher and the school. This was found to be especially true where telephonic contact was made. While it appeared to be the most convenient method, it was viewed as impersonal. Face-to-face contact and direct contact is reported to improve building a relationship with schools, principals, teachers, participants and the researcher (Galea & Tracy, 2007). Additionally, multimodal reminders may have been more effective with face-to-face contact as the primary method of contact (Galea & Tracy, 2007; Hartge, 2006; Keyzer et al., 2005). However, the methods of contact appeared to differ in each school. The research thus adapted according to the preference of the specific school.

In addition to the administrative challenges discussed, related to planning and scheduling, other challenges included relationships and consistency of researchers. These factors collectively affected the recruitment and dropout rates. Schools reported that the use of research assistants was inconsistent and reported that they were unable to build a relationship with and get to know the researcher and research assistant at their school. Building a relationship early on with the school is recommended by Galea and Tracy (2007) and Hartge (2006), as it has implications for data collection at future time intervals. Schools reported feeling that it was challenging to deal with different people and that they did not know who the sole contact person was. Schools further reported that if they had built a relationship with a consistent researcher, it may have been easier to make certain concessions where challenges around organisation and planning arose. Consequently, this affected their motivation and willingness to participate in this study, especially given the time constraints that the school faced. Given the demands that schools face and feedback that schools provided, it should be taken into consideration that data collection is an added responsibility taken on by the school.

### Preliminary estimates of treatment effect

Though no significant result was observed at 1 month post-intervention, it is possible that it was too early for participants to have internalised their learning. This is supported by Kathard et al. (2014), who stated that at 1 month post-intervention, an attitude shift was beginning but that more time was recommended and that 6 months post-intervention may yield further changes in treatment effect. In doing so, the results at 6 months post-intervention supported the use of the CCR at the 6-month interval in the construct of VI. This therefore illustrates that the use of the CCR intervention may facilitate a positive shift in the magnitude and direction of scores of attitudes towards CWS. The results suggest an indication of the direction of change in treatment effect. Despite the dropout of participants, the findings at 6 months show that evaluating treatment effect at 6 months post-intervention is a critical time period, as this is when the start of shift in treatment effect becomes apparent. The use of the CCR is important, as it may facilitate the holistic management of stuttering and communication difficulty by speech-language therapists (SLTs). It is important to note that while a potentially statistically significant result was observed at 6 months post-intervention within the VI construct of the SROM, this is not the sole finding to influence the feasibility of the RCT. It is repeatedly emphasised in the literature that collectively effectiveness of an intervention and procedural aspects are drawn upon to determine the feasibility of an RCT (Evans, 2003; Leon et al., 2011; Oakley et al., 2006; Shanyinde et al., 2011). This study is an illustration of this and has emphasised the need to draw on both components of this study to tell the researcher about how future planning may be facilitated.

## Conclusion

Overall, both procedural aspects and treatment effect trends provide important information about the feasibility of an RCT. It is illustrated that collectively these factors suggest that an RCT is feasible. The recruitment and dropout rates specifically showed that several factors should be considered to improve the feasibility of a future RCT in terms of the procedural aspects of this study. Additionally, the treatment effect shows that 6 months post-intervention proved to be an optimal and feasible time to determine the treatment effect, whereas in this study a significant result was noted only in one of the constructs at 6 months. It would be important to retain a sample to test the effectiveness of the CCR intervention in a more robust way. Furthermore, it would be impractical to measure post-intervention attitudes at three intervals in future because of time constraints (reported by schools) and because of the repeated use of the same outcomes measure.

### Strength, limitations and clinical implications

The main strength of this study was its ability to achieve the objectives of determining the feasibility of an RCT by drawing on the findings of this pilot study. The limitation of this study is the way in which schools experienced the study. Clinical implications include that an RCT is feasible and that there is a need for further research to enrich South African literature on classroom-based stuttering intervention.

### Recommendations for future research

An RCT is recommended, with further development of the process. In order to conduct a methodologically sound RCT, there are several factors that need to be considered and put into place, as described in the discussion. There are two main recommendations for this study: (1) to reduce the dropout rate of participants through stringent methods and (2) to determine treatment effect at baseline and 6 months post-intervention only. No significant results were noted at 1 month, suggesting that perhaps only 6 months post-intervention data may be necessary, as this is where the shift in treatment effect begins. By reducing the number of data collection intervals and being transparent about the number of visits that are required, the researcher may also alleviate time pressure and any burden schools may experience.
